# What are species and how are they formed?

**DOI:** 10.1093/nsr/nwad017

**Published:** 2023-02-09

**Affiliations:** Chung-I Wu School of Life Sciences, Sun Yat-Sen University, China

In this issue of *NSR*, four papers address various aspects of speciation. The fifth one, to appear in the next issue, is on biodiversity and should ultimately be about speciation as well. As has always been the case in speciation research, the conclusion depends on what ‘species’ are. This is true for Wang *et al.* [1] on post-speciation gene flow, Ma *et al.* [2] on hybridization speciation, Sun *et al.* [3] on sympatric speciation and Fan *et al.* [4] on species diversity. The conclusions would all be quite different if the species in question were not accepted as true species. The fifth one by Orr *et al.* [5] starts the series by tackling species delineation head on.

So, what are species? Ernst Mayr (1963) [6] proposed that species are objective entities, definable by reproductive isolation (RI). However, unless RI is defined as zero exchange of genes between species, it would not be an operationally usable criterion. For example, if only one of the two sexes is sterile, as is common between species, are these species deemed reproductively isolated? To be self-consistent, RI has to be a zero-tolerance concept [7–11]. With that strict criterion, many generally recognized species are no longer good species. Neither can strictly allopatric species be recognized as such because their RI status could not be determined.

The fundamental issue is whether there is a natural demarcation in the process of speciation that corresponds to an idealized species concept. The centrality of this issue is the legacy of Mayr, Coyne and Orr [12] who started their classic exploration of whether species are ‘realities of nature’. A false analogy we may make is the demarcation of seasons, which are definable by the solstices and equinoxes in relation to the solar declination. Such a demarcation is a physical reality independent of the observables, such as the weather. In contrast, species delineation may be more akin to human development, with no natural demarcation between childhood and adulthood. One could certainly suggest some physiological traits as the defining character. Such delineations, however, may be so narrow as to defy the general perception of adulthood, or species (full RI being a most obvious example).

While speciation is an objectively definable process, the stage where the diverging taxa are deemed species is a subjective decision [13,14]. The search for an objective and ideal system of species delineation has been unsuccessful [15,16] and may not see improvement in the future. In this issue, Orr *et al.* [5] provide a set of rules by which such subjective decisions should be made. Although their main point is about standardizing data collection and interpretation for taxonomic decisions, the significance is stated as follows:

‘taxonomists have been describing species for centuries (Costello 2022), so a universally accepted definition for species is clearly not a prerequisite for taxonomic research.’

This is a tacit recognition that species delineation and the concept of species can, or should, be decoupled. After all, naming species is a practical task that would enable people to accurately identify their subjects of interest. This task, as outlined in Orr *et al.* [5], has been charged to taxonomists for centuries, much like planetary scientists being in charge of defining and naming planets. This seems to be a reasonable proposition as long as taxonomists do it in a consistent manner. Consistency should be practiced by taxonomists and, as much as possible, deemed acceptable by other branches of biology. Species so delineated will be referred to as ‘taxonomic species’.

To achieve a degree of consistency, Orr *et al.* [5] propose six steps of data procurement and analysis. Whether the big-data approach can achieve the desired goal will have to be field tested. In particular, the fourth step on ‘species concept justification’ could be most challenging as there may be as much contention among taxonomists as in the larger community outside. Perhaps consistency in species delineation will have to take precedence over the alignment with some species concepts, of which there are many. Furthermore, it seems that some sort of weighting or parsing of different kinds of data will be needed. For example, different types of data may not be amenable to a coherent interpretation and, among different layers of molecular data, synthesis may not be possible.

Nevertheless, the six-step proposal itself is a bold first step toward decoupling species delineation and species concept. Such explicit criteria would inform us what these named species are and, no less important, what they are not. Furthermore, there will be only one species-naming system even though there are still 32 species concepts [17–19].

As current species delineations seem somewhat arbitrary, species are often interpreted to suit different needs for different people. A standardized naming system would require biologists to provide species information pertinent to their studies that is not in the consideration of the naming system. Too often, taxonomic species are conflated with other delineations, such as the biological species concept (or BSC). As a result, taxonomic species may be assumed to be reproductively isolated even though RI is not part of the delineation criteria. Thus, it becomes even more important that the taxonomic methods used and their underlying lines of evidence be made clear in future taxonomic work.

The three articles of genomic analyses (Wang *et al.* [1]; Sun *et al.* [3]; Ma *et al.* [2]) treat the named species differently in relation to their own research objective. Wang *et al.* [1] are able to avoid the uncertainty of species status by using what they call the ‘secondary sympatry test’, which is a classical and most reliable criterion for species delineation. In this test, two mangrove species (*Rhizophora stylosa* and *Rhizophora mucronata*) [20,21] have diverged in allopatry and become sympatric later on (hence, secondary sympatry). During prolonged sympatry, they remain distinct species, as observed in northern Australia, even with ample opportunities over thousands of years to merge into one hybrid swarm.

Given the two species that have passed the test, Wang *et al.* [1] report a most unusual pattern of gene introgressions between species. They observed that genetic exchanges continued over a long period of sympatry and thus resulted in a very fine pattern of gene introgressions, averaging at 5–10 kb. They also noted that the timing of establishing the secondary sympatry is crucial for observing such fine-grained introgressions. If it is too early, a hybrid swarm would be the outcome, but if it is too late, there would be no introgressions.

While ‘speciation with gene flow’ has been regularly reported [7,22–27], these papers collectively do not challenge the BSC because the reported gene flow most likely happened in the early stages of speciation [7,22–26,28–32]. After all, most geographical isolation develops gradually. For example, it took 3 million years for the Panama Isthmus to form and gene flow should be common in the early stages. To satisfy the conditions of the BSC, it would only take a period of zero gene flow at the conclusion of the process of speciation. The BSC does not stipulate how speciation starts, only how it concludes. In short, Wang *et al.* [1] may be the first study that truly establishes post-speciation gene flow which, of equal importance, persisted for a long period.

Studying the other end of the process right at the beginning of speciation, Sun *et al.* [3] focus on the possibility of sympatric speciation between fishes of a glacier lake. While it may appear to be a typical (albeit not easily accessible) case of sympatric speciation, their conclusion is not typical at all. They propose that this type of sympatric speciation may be micro-allopatric or micro-parapatric speciation. Here, they make an uncommon, but not unreasonable, proposal that the geographical scale should be measured by the opportunities of gene flow rather than by the physical scale. In sympatry, gene flow should be unimpeded; hence, the presence of local barriers to gene flow may often invalidate the sympatry assumptions.

To measure the extent of gene flow during speciation, the authors surveyed the genomes of the two incipient species and observed many large genomic segments that were significantly more divergent than the rest of the genomes. Such segments are often referred to as ‘genomic islands’ [14,33–38], which can be hundreds of kb in size in the fishes they study. In contrast, with unimpeded gene flow in sympatry, the genomic islands ought to be very small. In their simulations using values from a limited parameter space, the authors claim that the genomic islands should be of the gene size, if not smaller. They also cite Wang *et al.* [1], who report very fine genomic admixture in secondary sympatry, mostly in the size range of individual genes.

Sun *et al.* [3] hence reject the model of sympatric speciation between the fishes. They suggest that, even in a topologically simple glacier lake, there is sufficient environmental heterogeneity (such as different depths of the water column) for micro-allopatry or micro-parapatry. The study leaves two questions for further debate. The first is that sympatry is still a possible explanation if sexual isolation is the mechanism of RI. It is not a coincidence that most theoretical studies [39–43] show that sympatric speciation is feasible under (and mainly under) sexual isolation. Sun *et al.* [3] report spatial segregation as the mechanism of isolation without ruling out sexual isolation as the primary mechanism. The second question is, if not sympatry, what is it? Is it micro-allopatry or micro-parapatry? This issue is the classical allopatry vs. parapatry debate writ small and has been debated recently [7–11]. The key conclusion of Sun *et al.* [3], nevertheless, is that the appearance of sympatric speciation may not be truly sympatric at the micro-geographical scale.

A third genetic study of this issue is by Ma *et al.* [2] on hybridization speciation of *Rhododendron.* The impressive diversity of *Rhododendron* in the Hengduan mountains in southwestern China is akin to the cichlid fishes of Lake Victoria [44–46]. Among the papers of this issue, the interpretations here are most strongly dependent on the delineation of species. In this study, the authors investigated 238 ‘taxonomic species’ that were named by botanist-taxonomists throughout most of the 20th century in China on the basis of morphology and zonation.

Ma *et al.* [2] report extensive introgressions and hybridizations among these taxonomic species. New species often emerge via ‘hybridization speciation’. Such extensive admixture is reflected in the strong overlaps between interspecific and intraspecific diversity (2.3–8.7 × 10^−3^ vs. 0.9–8.2 × 10^−3^, respectively) in this amazing genus. In their interpretation, a possible controversy would be whether the taxonomic species of *Rhododendron* would correspond to the general concept of species. If we use the stringent biological species concept that demands full RI, the answer would be ‘No’. While hybridization speciation may occasionally be observed, extensive hybridizations as reported here may in fact indicate two fully reproductively compatible parents. Instead of multiple species, it can be argued that these are varieties in a polytypic species. Most domestic plants (such as many crops) and animals (e.g. dogs with multiple breeds) are polytypic species capable of yielding even more varieties via hybridization. One may even argue that *Homo sapiens* is also a polytypic species. Nevertheless, the authors can reasonably contend that the taxonomic species are valid species as well. They are just not species according to the BSC. After all, the BSC has many deficiencies, both conceptually and operationally, and should more appropriately be referred to as the ISC (i.e. isolation species concept [7,14]).

The interesting biological issue should not be whether the taxonomic species of *Rhododendron* are species. Instead, the evolution of *Rhododendron* affirms the common belief in the power of hybridization between separately evolving lineages, before they become reproductively incompatible. We do see such power in domesticated plants and animals in their richly diverse forms. The ‘power of hybridization’ is equivalent to the mixing-isolation-mixing (MIM) model of speciation [47] whereby the genetic diversity is stored up in isolation and mixed (then recombined and selected) in the mixing phase. In this sense, the MIM model is extended to the post-speciation phase, with species defined as in Ma *et al.* [2]. In theory, species number in the MIM model can increase exponentially, and He *et al.* use this model to explain the exceedingly high level of biodiversity in the Indo-western Pacific (IWP). This is also the topic of the last of the five papers.

Fan *et al.* [4] analyze global marine biodiversity, which is highest in the IWP. The objective of Fan *et al.* [4] is not only about the distribution of biodiversity but is also concerned with conservation decisions. They declare that strategically protecting ∼22% of the ocean would allow us to reach the target of conserving ∼95% of currently known taxonomic, genetic and phylogenetic diversity. Against this backdrop, the species question could have been an even thornier issue in conservation efforts, which must be spread over as many species as possible.

Fan *et al.* [4] adopt a conservation idea of nearly 30 years, based on ‘phylogenetic diversity’ [48–53] and extend it in two ways. They incorporate the evenness of the phylogeny (i.e. rare and distant lineages given a higher weight) and within-species genetic diversity. In such a framework, biodiversity is a continuum of diversity accumulation as the taxa evolve. Conservation efforts are geared toward maximizing the total genetic divergence in the assemblage of taxa of concern, rather than maximizing the number of species. In this approach, species delineation, embedded in the continuum, does not stand out as an intractable problem.

Applying this general framework of phylogenetic diversity, Fan *et al.* [4] highlight many marine regions globally most deserving of conservation efforts. The conservation proposal seems a brilliant idea but there is a caveat. Phylogenetic distances, like all DNA-based distances, are more a measure of evolutionary time (calibrated against mutation rate) than an indication of phenotypic or functional divergence. Humans and chimpanzees are divergent at the level of 1.1% which is lower than the within-species diversity of many other species. Certainly, no species has a level of phenotypic diversity as high as that between humans and chimpanzees, even though genetic diversity in the human-chimpanzee duo is quite modest. For this reason, the approach of phylogenetic distance should be considered a brilliant step toward biodiversity conservation, using genomic data. The ultimate goal may be to conserve phenotypic and functional diversity.

In recent years, there has been reluctance to address the species question. The reluctance, however, cannot bridge the gulf between species designation and species concept. With the confusion, biologists could assume the named species (in their own study or studies they consult) to have the properties of a certain species concept. For example, Wang *et al.* [1] discuss the difficulties in finding evidence for post-speciation gene flow whereas many may argue that such evidence is everywhere. Certainly, if one uses rather loose criteria to delineate species, post-speciation gene flow ought to be common. In fact, one may say that Ma *et al.*’s report [2] is evidence for massive post-speciation gene flow. In contrast, one could also argue that hybridization speciation presented by Ma *et al.* [2] is not about species but about polytypes within a larger species. Clearly, species can have rather different meanings as evident in these two studies as well as in Sun *et al.* [3] and Fan *et al.* [4]. The next paragraph justifies the coexistence of diverse meanings of species.

Speciation is the transition process from one ancestral to two distinct descendant species. The extensive genic divergence between sibling species [33,54–56], or even between racial groups of the same species [57,58], indicates that the transition is gradual and genetically complex. For that reason, the demarcation of species can be set anywhere during that transition. This transition is unlike physical phenomena such as seasons, defined by solstices and equinox. Demarcation in different speciation processes may be placed differently depending on the biological properties of interest.

The standardization of taxonomic species (Orr *et al.* [5]) will be a first step toward eliminating the confusion over what the named species are. Taxonomic species will follow a set of rules that are likely to be incompatible with many species concepts. Investigators will have to state exactly how species are delineated in their study, as has been done in each of the papers in this issue. A large set of publications in a forthcoming book on speciation (Cold Spring Harbor, *Perspectives in Speciation*, editors: Catherine L. Peichel, Daniel I. Bolnick, Åke Brännström, Ulf Dieckmann and Rebecca J. Safran, forthcoming 2023) should bring further attention to speciation and species delineation.
